# Assessing the association between occupancy and outcome in critically Ill hospitalized patients with sepsis

**DOI:** 10.1186/s12873-015-0049-y

**Published:** 2015-10-19

**Authors:** Dean W. Yergens, William A. Ghali, Peter D. Faris, Hude Quan, Rachel J. Jolley, Christopher J. Doig

**Affiliations:** Department of Community Health Sciences, University of Calgary, Calgary, Canada; Department of Critical Care Medicine, University of Calgary, Calgary, Canada; Department of Internal Medicine, University of Calgary, Calgary, Canada; Snyder Institute for Chronic Disease, Faculty of Medicine, University of Calgary, Calgary, Canada; Institute for Public Health, Faculty of Medicine, University of Calgary, Calgary, Canada

**Keywords:** Patient flow, Occupancy, Admissions, Health services research, Sepsis, Emergency department, Intensive care unit

## Abstract

**Background:**

Sepsis has a high prevalence, mortality-rate and cost. Sepsis patients usually enter the hospital through the Emergency Department (ED). Process or structural issues related to care may affect outcome.

**Methods:**

Multi-centered retrospective observational cohort study using administrative databases to identify adult patients (> = 18 years) with sepsis and severe sepsis admitted to Alberta Health Services Calgary zone adult multisystem intensive care units (ICU) through the ED between January 1, 2006 and September 30, 2009. We examined the association between ICU occupancy and hospital outcome. We explored other associations of hospital outcome including the effect of ED wait time, admission from ED during weekdays versus weekends and ED admission during the day versus at night.

**Results:**

One thousand and seven hundred seventy patients were admitted to hospital via ED, 1036 (58.5 %) with sepsis and 734 (41.5 %) with severe sepsis. In patients with sepsis, ICU occupancy > 90 % was associated with an increase in hospital mortality even after adjusting for age, sex, triage level, Charlson index, time of first ED physician assessment and ICU admission. No differences in hospital mortality were found for patients who waited more than 7 h, were admitted during the day versus night or weekdays versus weekends.

**Conclusions:**

In patients with sepsis admitted via the ED, increased ICU occupancy was associated with higher in-hospital mortality.

## Background

Sepsis is an important issue in the hospital, especially in the Intensive Care Unit (ICU), due to its prevalence, mortality, and the healthcare resources used in caring for these patients. In the United States, there are 750,000 sepsis cases annually with crude mortality ranging from 30 to 50 % [1, 2] and is the leading cause of death in non-coronary ICU [3]. In Canada, 30,587 sepsis hospitalizations occurred in 2008–2009 (outside of Quebec) [4] and in-hospital mortality was reported at 38.1 % [5]. Treating sepsis is expensive, costing as much as $50,000 per patient, or an estimated annual cost of $17 billion in the United States [6]. In Canada, the cost for caring for sepsis patients is estimated at $325 million per year [5].

Caring for sepsis patients puts a tremendous strain on the healthcare system, especially around some of the most critical resources such as the emergency department (ED) and the ICU. Previous studies have found that 55 to 60 % of all sepsis patients are admitted through the ED [2, 3, 7, 8]. Sepsis is increasing in the general population with severe sepsis hospitalizations having doubled over an 11 year period [9]. To combat the high mortality of sepsis, several international intervention programs have been developed in an effort to improve the treatment and outcome of sepsis and severe sepsis patients. One of the larger and more successful of these intervention programs is the Surviving Sepsis Campaign (SSC) [10]. The SSC focused on the rapid identification of infection, early antibiotic administration, and rapid resuscitation starting in the ED. Previous research has demonstrated ED crowding may delay early treatment of infections, and increased risk of medication errors, both of which may be associated with a worse outcome [11, 12].

Donabedian [13] in addressing quality assurance in healthcare describes a model that consists of outcome, process and structure. Both process and structure contribute to the outcome of the patient. In examining this approach, process may influence the outcome of sepsis patients through a variety of mechanisms such as the ability to diagnose a patient in a timely manner according to established guidelines, as well as, having the process in place to hand off the patient to the appropriate level of care, such as between the ED and the ICU. Structurally, resources such as appropriate availability of beds must also be present so that a patient’s treatment is not delayed or adversely affected resulting in a negative outcome.

The primary purpose of this paper is to explore the association between ICU occupancy (an important Donabedian structure element) and outcomes of hospital stay in patients with sepsis. Our study hypothesis was that ICU occupancy would not be associated with a differential outcome, but that admission during night or weekend would be associated with a worse outcome. We also used a rich combination of linked databases to explore secondary questions focusing on other structure and process variables (ED wait time, admission on weekday versus weekends and admission on days versus nights) that may influence outcomes.

## Materials and methods

### Study population

This was a multi-site retrospective observational cohort study consisting of adult patients with sepsis admitted to the Foothills Medical Centre (FMC), Peter Lougheed Centre (PLC) or Rockyview General Hospital (RGH) through the ED between January 1, 2006 and September 30, 2009 in the Calgary zone of Alberta Health Services (AHS). The Calgary zone of Alberta Health Services provides virtually all acute hospital care to the residents of the cities of Calgary and Airdrie and surrounding communities in the province of Alberta, Canada.

Sepsis and severe sepsis were defined as described in the Canadian Institute for Health Information "In Focus: A National Look at Sepsis" [4]. Sepsis was defined as having one of the following ICD-10-CA codes: A03.9, A02.1, A20.7, A21.7, A22.7, A23.9, A24.1, A26.7, A28.0, A28.2, A32.7, A39.2, A39.3, A39.4, A40.–, A41.–, A42.7, B00.7, B37.7, P36.–, P35.2, P37.2 and P37.5. The diagnosis type was modified to only include the main diagnosis (M), pre-admission comorbidity (1) or second pre-admission comorbidity (3) to better identify patients with sepsis at the time of hospital admission. Additional information on ICD-10-CA codes can be referenced in the International Statistical Classification of Diseases and Related Problems documentation [14]. Severe sepsis was defined as sepsis with the addition of organ dysfunction occurring in at least one of the following six systems from the following associated ICD-10-CA codes, Hematologic D69.5, D69.6, D65; Cardiovascular R57.–, I95.1, I95.8, I95.9; Hepatic K72.0, K72.9, K76.3; Neurologic F05.0, F05.9, G93.1, G93.4, G93.80; Renal N17.–; and Respiratory J96.0, J96.9, J80, R09.2.

### Data sources

The study population was extracted from three administrative databases by a trained and experienced health analyst within Alberta Health Services independent of the investigators. These databases were the Inpatient Discharge Abstract Database (DAD) which includes all Health Records related information such as ICD10 diagnosis and in-hospital outcome, the Admission, Discharge, Transfer (ADT) database which includes all physical bed-management patient transfers in the acute care hospital and the Ambulatory Care Classification System database (ACCS) which provides information on ED patient encounters in the acute care hospitals. The DAD system, which provides the coded ICD10 diagnosis data extracted from the patient’s chart, is maintained by professionally trained health records coders and has been described previously [15].

Each database had a common unique patient identifier. All data were collected following removal of any identifiable information such as name or address. The three databases were then linked together using the patient identifier with the hospital date of admission, and following data linkage, the individual patient identifier was encrypted to ensure anonymity. All databases were imported into a Postgres relational database (http://postgresql.org) for data management and the creation of the study population. The Postgres database was then interfaced to the statistical software, R version 2.10 (http://r-project.org), through ODBC (Open Data Base Connectivity) for the statistical analysis.

### Operational definitions

The ACCS database had detailed information about the patient's ED visit. This data included time of arrival, triage level using the CTAS (Canadian Triage and Acuity Scale) score [16] determined in accordance with their published guidelines, and time of first ED physician assessment. The ED wait time used in our analysis was dichotomized as less than or equal to 7 h or greater than 7 h; this variable was calculated as the difference from when the patient was admitted to the ED and the time that the patient was admitted to the hospital.

The ADT database included information on patient movement (flow) including time stamps for admission/discharge/transfer into the hospital and all units throughout the hospital. The time stamps are automatically generated through a bed management system linked by an HL7 interface engine to the clinical information systems. ICU occupancy was calculated from the ADT system at the time of first ED physician assessment assuming that the physician was the most likely factor to determine the need for admission.

Outcome was extracted from the DAD database, and was defined as either all cause in hospital mortality during the same admission or discharge alive irrespective of location that the patient was discharged to (i.e. home, convalescent care, et cetera). The DAD was also used to define sepsis and severe sepsis as described above. We applied the ICD-10 coding algorithm to the DAD using the methods described by Quan et al. [17], and previously applied in critically ill patients [18] to determine the Charlson index score [19].

### Statistical analysis

Analysis was performed using R version 2.14 (www.r-project.org). Normally or near normally distributed data were reported as Mean ± SD and any comparisons used the Student *t* test. Non-normally distributed data were reported using the Median and Interquartile Range (IQR) and any comparisons used the Mann-Whitney *U* test. Categorical data were assessed using Fisher exact test for pair wise comparisons. Logistic regression models were developed to examine the effect of independent risk factors on in-hospital mortality.

Ethics approval was obtained from the Conjoint Health Research Ethics Board at the University of Calgary. Individual consent was not required as this was a cohort study with a large number of deaths (obtaining consent not practical) and all data made available to the investigators was anonymized.

## Results

### Study population

**Fig. 1:**
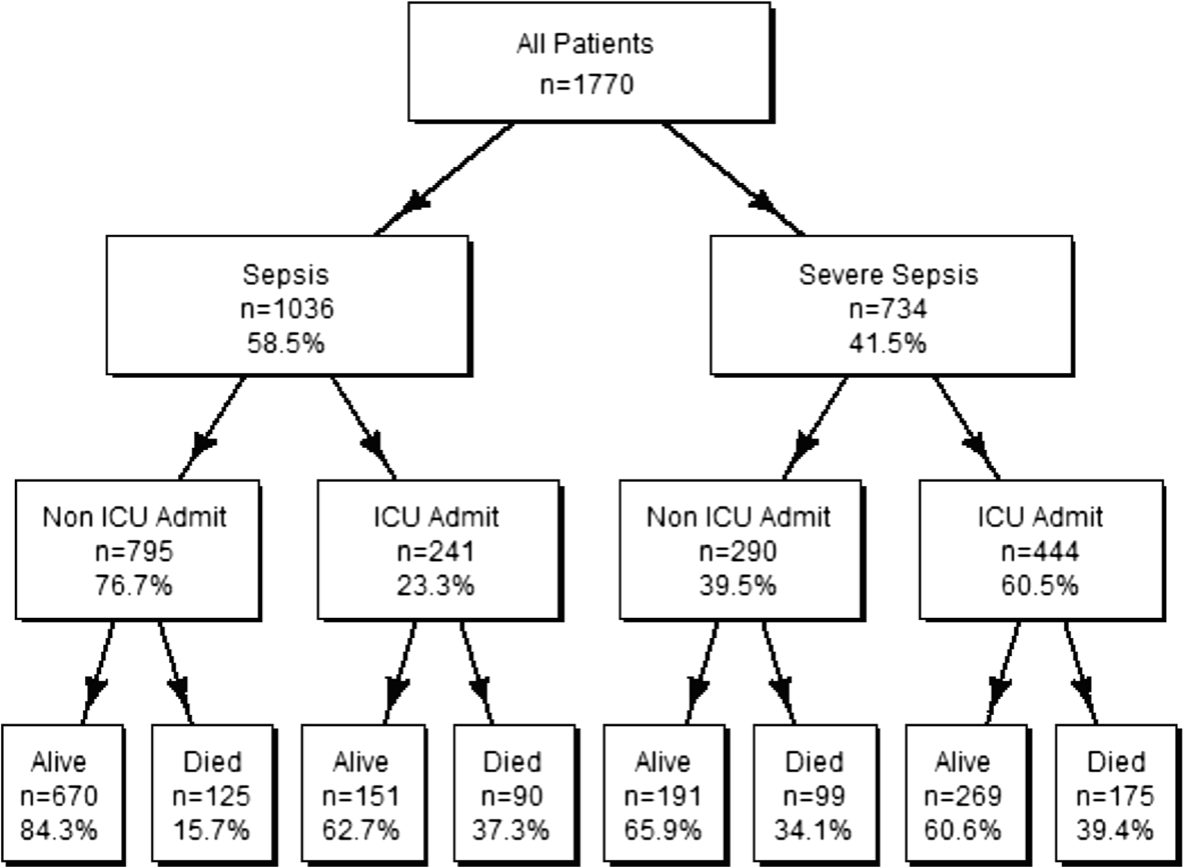
Breakdown of diagnosis, admission to ICU and in-hospital mortality for the study population

There were 1770 sepsis or severe sepsis admissions through the ED during the study period (Fig. 1). Table 1 describes the characteristics of patients by sepsis and severe sepsis strata. From these patients, 1036 (58.5 %) were categorized as having sepsis while 734 (41.5 %) had severe sepsis. From all patients, there were 812 (45.9 %) female patients versus 958 (54.1 %) male patients. 241 out of 1036 (23.3 %) sepsis patients were admitted to the ICU, whereas 444 out of 734 (60.5 %) severe sepsis patients required care in the ICU. The median age of the patients was 65.8 years (IQR: 52.3, 78.9). The patients had a median ED length of stay (LOS) of 11 h (IQR: 6,18), median ICU LOS of 5 days (IQR: 2,10), and a median hospital LOS of 10 days (IQR: 5,22). Sixty four point five percent (64.5 %) of patients had a Charlson index score of 0 (no chronic health conditions), 29.3 % had scored a 1 or 2 and 6.2 % had a Charlson index score of 3 or more. The number of patients discharged alive was 1281 (72.4 %) and 489 (27.6 %) died in hospital.

**Table 1:** Patients admitted to hospital via emergency department

	All patients	Sepsis patients	Severe sepsis patients
Variable	*n* = 1770	*n* = 1036 (58.5 %)	*n* = 734 (41.5 %)
Age (years) Median (IQR)	65.75 (52.25,78.92)	65.71 (51.67,79.27)	65.83 (53.25,78.06)
Sex (female)	812 (45.9 %)	478 (46.1 %)	334 (45.5 %)
Site			
Foothills medical center	652 (36.8 %)	354 (34.2 %)	298 (40.6 %)
Peter lougheed hospital	608 (34.4 %)	343 (33.1 %)	265 (36.1 %)
Rockyview general hospital	510 (28.8 %)	339 (32.7 %)	171 (23.3 %)
ED LOS (hours) Median (IQR)	11 (6,18)	12 (7,19)	9 (5,15)
ICU LOS (days) Median (IQR)	5 (2,10)	4 (2,8)	5 (2,11)
Hospital LOS (days) Median (IQR)	10 (5,22)	8.5 (4,18)	12 (5.25,26)
Bed transfers mean ± SD	2.8 ± 2.44	2.47 ± 2.13	3.27 ± 2.76
Triage Level			
CTAS 1-Resucitation	137 (7.7 %)	42 (4.1 %)	95 (12.9 %)
CTAS 2-Emergent	1075 (60.7 %)	624 (60.2 %)	451 (61.4 %)
CTAS 3-Urgent	540 (30.5 %)	359 (34.7 %)	181 (24.7 %)
CTAS 4-Less urgent	17 (1 %)	10 (1 %)	7 (1 %)
CTAS 5-Non urgent	1 (0.1 %)	1 (0.1 %)	0 (0 %)
Charlson index score			
0	1142 (64.5 %)	653 (63 %)	489 (66.6 %)
1 or 2	519 (29.3 %)	333 (32.1 %)	186 (25.3 %)
3 or more	109 (6.2 %)	50 (4.8 %)	59 (8 %)
ICU admission (admitted)	685 (38.7 %)	241 (23.3 %)	444 (60.5 %)
Hospital outcome (alive)	1281 (72.4 %)	821 (79.2 %)	460 (62.7 %)

### Relationship of ICU occupancy to outcome

Table 2 shows sepsis patient characteristics and outcome stratified by ICU occupancy. A gradual decrease was observed for hospital survival as ICU occupancy increased with 80.8, 78.9, 78.8 and 72.6 % of sepsis patients being discharged alive as associated with ICU occupancy of less than 80 %, 80–84 %, 85–89 % and 90 % and above. No other clinically relevant differences were observed. Table 3 shows the characteristics and outcome stratified by ICU occupancy for severe sepsis patients. No similar decrease in hospital survival as ICU occupancy increased was observed.

**Table 2:** Sepsis patients admitted examining ICU occupancy

	ICU occupancy	ICU occupancy	ICU occupancy	ICU occupancy
	× < 80 %	80 % ≤ × < 85 %	85 % ≤ × < 90 %	90 % < ×
Variable	*n* = 595 (57.4 %)	*n* = 204 (19.7 %)	*n* = 113 (10.9 %)	*n* = 124 (12 %)
Age (years) Median (IQR)	65.58 (51.71–79.96)	64.08 (48.63–75.77)	69 (56.08–79.75)	66 (51.31–78.83)
Sex (female)	278 (46.7 %)	89 (43.6 %)	52 (46 %)	59 (47.6 %)
ED LOS (hours) Median (IQR)	11 (7–19)	12.5 (8–19)	12 (8–19)	12 (7–20)
ICU LOS (days) Median (IQR)	4 (2–7)	3 (1–8.75)	3 (2–6.5)	4 (2–11)
Hospital LOS (days) Median (IQR)	8 (4–16)	9 (4–23)	10 (4–18)	9 (4–18)
Bed transfers mean ± SD	2.33 ± 1.86	2.5 ± 2.2	2.76 ± 2.25	2.81 ± 2.95
Triage level mean ± SD	2.35 ± 0.58	2.32 ± 0.55	2.31 ± 0.55	2.24 ± 0.56
Charlson index mean ± SD	0.55 ± 1.09	0.76 ± 1.3	0.54 ± 0.77	0.58 ± 1.09
ICU admission (Admitted)	134 (22.5 %)	50 (24.5 %)	27 (23.9 %)	30 (24.2 %)
Hospital outcome (alive)	481 (80.8 %)	161 (78.9 %)	89 (78.8 %)	90 (72.6 %)

**Table 3:** Severe sepsis patients admitted examining ICU occupancy

	ICU occupancy	ICU occupancy	ICU occupancy	ICU occupancy
	× < 80 %	80 % ≤ × < 85 %	85 % ≤ × < 90 %	90 % < ×
Variable	*n* = 392 (53.4 %)	*n* = 159 (21.7 %)	*n* = 84 (11.4 %)	*n* = 99 (13.5 %)
Age (years) Median (IQR)	65.83 (53.31–77.56)	66.83 (55.25–78.25)	63.67 (49.75–77.5)	65.83 (53.96–78.08)
Sex (female)	172 (43.9 %)	72 (45.3 %)	45 (53.6 %)	45 (45.5 %)
ED LOS (hours) Median (IQR)	8 (5–14.25)	9 (4–15)	11 (6–19)	9 (5.5–15.5)
ICU LOS (days) Median (IQR)	5 (2–9)	7 (3–15.25)	6 (2.25–15.25)	6 (2–9)
Hospital LOS (days) Median (IQR)	11 (6–23)	14 (6.5–28)	16 (5–33.25)	12 (5–34)
Bed transfers mean ± SD	3.03 ± 2.43	3.7 ± 3.48	3.32 ± 2.11	3.52 ± 3.1
Triage level mean ± SD	2.11 ± 0.67	2.14 ± 0.59	2.18 ± 0.58	2.18 ± 0.58
Charlson index mean ± SD	0.55 ± 0.98	0.66 ± 1.29	0.94 ± 1.62	0.45 ± 0.85
ICU admission (Admitted)	233 (59.4 %)	100 (62.9 %)	54 (64.3 %)	57 (57.6 %)
Hospital outcome (alive)	245 (62.5 %)	99 (62.3 %)	50 (59.5 %)	66 (66.7 %)

**Fig. 2:**
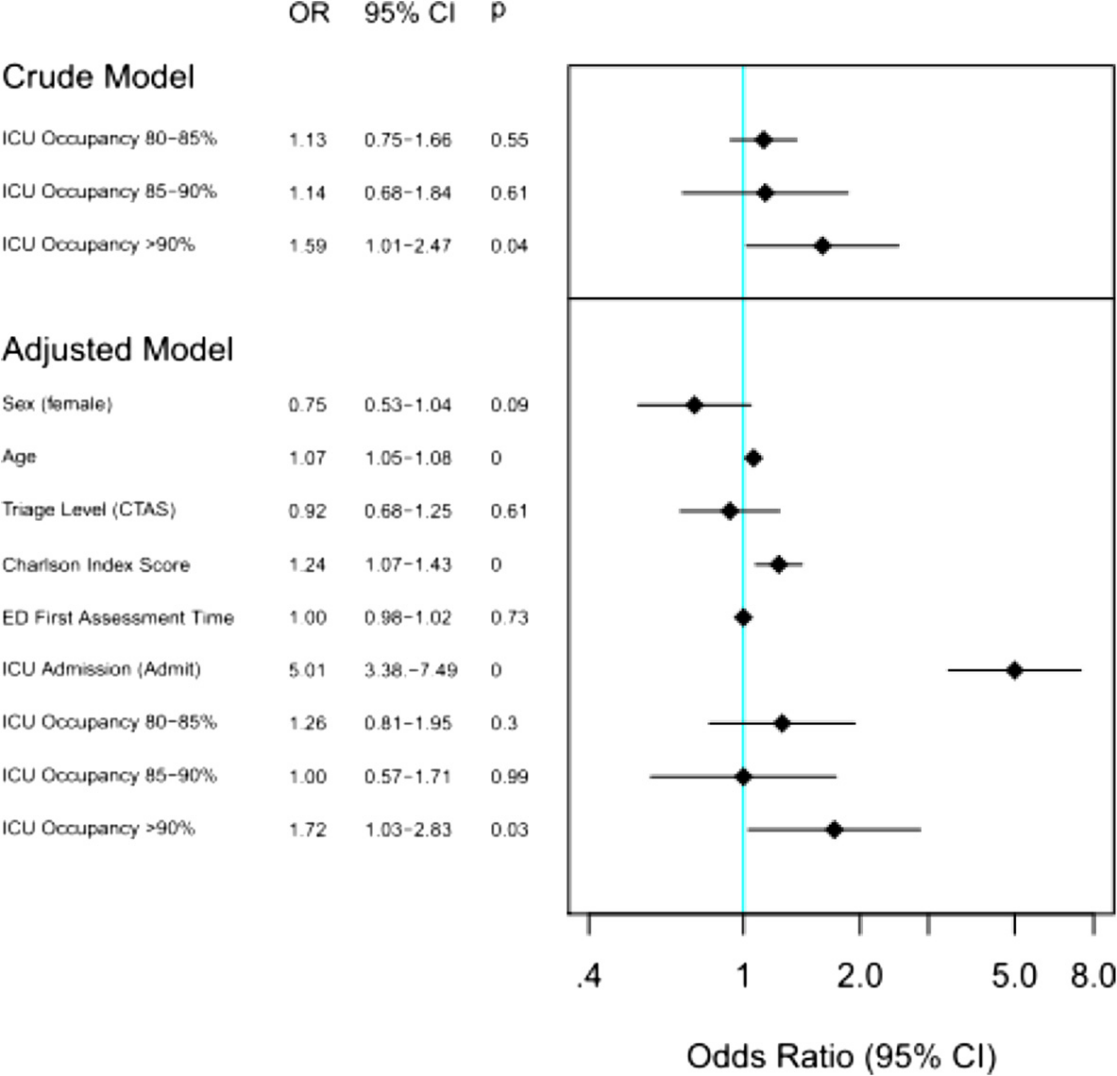
Logistic regression modeling of factors associated with in-hospital death among sepsis patients admitted via the emergency department

A multivariable logistic regression model was developed to examine the factors associated with in-hospital death for sepsis patients admitted via the ED and its association with ICU occupancy (Fig. 2). After controlling for sex, age, triage level, Charlson index score, ED Physician first assessment time and admission to ICU we found that increased ICU occupancy was statistically significant and a major risk factor for in-hospital mortality.

No statistically significant differences were found when examining patients with severe sepsis and ICU occupancy.

### Relationship of ED length of stay to outcome

The length of stay in the ED was examined to compare patients who waited less than (<=) 7 h versus patients who waited more than (>) 7 h. Seven hours was chosen a priori after consultation with local clinical experts as a time period which should reasonably permit the ED staff to initiate goal directed resuscitation, to administer the first dose of antibiotics, to assess clinical response, and to then decide the disposition of the patient’s admission. Table 4 describes the patient characteristics stratified by ED length of stay. Across both groups (sepsis and severe sepsis), patients waiting less than 7 h were younger than those who waited longer. In all groups, there was also a corresponding statistically significant difference with the ED triage level.

**Table 4:** Emergency wait less than or greater than 7 h

	All patients			Sepsis patients			Severe sepsis patients		
	*n* = 1770			*n* = 1036 (58.5 %)			*n* = 734 (41.5 %)		
	≤7 h	>7 h	P	≤7 h	>7 h	P	≤7 h	>7 h	P
Variable	*n* = 488 (27.6 %)	*n* = 1282 (72.4 %)		*n* = 226 (21.8 %)	*n* = 810 (78.2 %)		*n* = 262 (35.7 %)	*n* = 472 (64.3 %)	
Age (years) Median	60.83	67.67	< 0.001	60.58	67.17	*0.001*	61	68.21	< 0.001
Sex (female)	213 (43.6 %)	599 (46.7 %)	0.268	104 (46 %)	374 (42.2 %)	0.973	109 (41.6 %)	374 (46.2 %)	0.133
ICU LOS (days) Median	5	4	0.974	3	4	0.59	5	5	0.946
Hospital LOS (days) Median	10	10	0.693	7.5	9	0.082	13.5	11	0.337
Bed transfers mean	3.08	2.7	*0.008*	2.59	2.43	0.38	3.5	3.15	0.115
Ward transfers mean	1.9	1.71	*0.007*	1.67	1.57	0.26	2.1	1.95	0.183
Triage level mean	1.94	2.37	< 0.001	2.08	2.4	< 0.001	1.82	2.31	< 0.001
Charlson index mean	0.6	0.6	0.978	0.56	0.6	0.652	0.63	0.59	0.639
ICU admission (admitted)	323 (66.2 %)	362 (28.2 %)	< 0.001	99 (43.8 %)	142 (17.5 %)	< 0.001	224 (85.5 %)	220 (46.6 %)	< 0.001
Hospital outcome (alive)	324 (66.4 %)	957 (74.6 %)	0.001	169 (74.8 %)	652 (80.5 %)	0.075	155 (59.2 %)	305 (64.6 %)	0.166

Patients who were triaged as the sickest (based upon CTAS score) had the quickest hospital admission. Also patients who required care in the ICU in both groups (sepsis and severe sepsis) were more likely to be admitted within 7 h (66.2 %) compared to those admitted after 7 h (28.2 %). Only for the all patient group was there a statistically significant difference for in-hospital mortality with 66.4 % of patients waiting less than 7 h leaving the hospital alive compared to 74.6 % for those waiting longer than 7 h.

### Relationship of ED admission day/hour to outcome

We looked at all sepsis patients admitted to the ED during the day (*n* = 1462) defined as 07:00 am to 10:59 pm compared to patients admitted at night (*n* = 308) defined as 11:00 pm to 06:59 am. Only one important difference was found, patients admitted during the day were less likely to be admitted to the ICU (37.2 %) as compared to patients admitted at night (45.8 %).

We looked at patients admitted from the ED during a weekday (*n* = 1300) defined as Monday to Friday compared to patients admitted during the weekend (*n* = 470) defined as Saturday or Sunday. There were no statistically significant differences found.

## Discussion

Our study examined the outcome of patients with sepsis and severe sepsis admitted through the ED and ICU occupancy. A higher mortality was observed in sepsis patients when an increased ICU occupancy was observed. After conducting a multivariate logistic regression it appears that an ICU occupancy > = 90 % at the time of physician assessment in the ED was the most significant risk factor associated with an increased in-hospital mortality for sepsis patients. Our data is consistent with the observations by Stelfox et al. [20] who identified that the number of ICU beds at the time of an acute deterioration in a hospitalized patient affected process of care, and that the number of ICU beds available was associated with these critically ill patients being less likely to be admitted to ICU.

The finding of an association between an increase in ICU occupancy and higher in-hospital mortality presents an interesting avenue for quality assessment for ICU's that have high occupancy rates. A plausible explanation for this occurrence is that for severe sepsis patients presenting in the ED, the severe nature of the disease necessitates that the ICU resources are freed up even in times of limited capacity. However, in a patient with sepsis who is less severely ill at presentation, a decision might be made based on knowledge of the ICU occupancy, for the patient to be admitted to a ward hoping that the patient will ‘do okay’. If this is a plausible explanation, our data suggests that this attempt to compromise when faced with a conflict of individual patient best interest and organizational capacity might result in patient harm. The sepsis patient may be in a care setting that is not capable of adequately treating or monitoring their condition, and their individual care is compromised resulting in a worse outcome. The hospital environments in our study contained no high observation units (immediate care settings) that would act as an adjunct or alternative care setting to the ICU.

The Donabedian quality assessment framework tries to make a distinction between suboptimal care which is a consequence of structural problems in contrast to suboptimal care which arises as a consequence of process of care issues. If the reason not to admit a patient is simply due to a lack of bed availability, then as a structural problem in delivering care, an appropriate response would be to increase the number of ICU beds. Studies examining the association between mortality and ICU occupancy have been conducted [21, 22]. In one study, an association was found between mortality and ICU occupancy (for example an odds-ratio of 1.3 for mortality and occupancy). These studies were not specific to patients with sepsis, and examined the outcomes for patients already admitted to the ICU. However, if any suboptimal care was due to a lack of knowledge or skills on the non-ICU ward to care for the sepsis patient, the appropriate response to these findings may be to address knowledge or care deficiencies on the ward. There has been published evidence [23] that nursing staff on non-ICU wards may have difficulty recognizing signs of sepsis, particularly when a patient is unwell or does not have an established diagnosis. Furthermore, there was a general lack of awareness for signs of organ dysfunction (such as elevated lactate levels or a low systolic blood pressure) indicative of development of septic shock.

Several limitations were encountered in this study. First, the Discharge Abstract Database (DAD) has no timestamps associated with the diagnosis fields impacting our ability to firmly establish when the patient was first diagnosed as having sepsis. To deal with this, ICD diagnosis type codes of main diagnosis (M), pre-admission comorbidity (1) and second pre-admission comorbidity (3) were utilized. Second, the ADT database contains patient specific bed location by timestamps, however, does not contain information related to available beds such as staffing availability or ratios. However, given that our data was collected over 2.5 years, we have assumed that any variability in staffing ratios/availability were sources of random error. During the interval of our study, all ICU beds in each of the 3 units were always fully staffed (i.e. open), however, we cannot be assured that the same was true with respect to overall hospital beds.

## Conclusions

In conclusion, we have shown that there is an association between higher ICU occupancy rates and an increase in in-hospital mortality for sepsis patients. Given the prevalence and increasing incidence associated with general aging of the population, the high mortality rates, and the cost, further research should be considered to determine if this association is a process issue, such as the misidentification of border-line sepsis patients needing additional attention or lack of awareness of signs of clinical deterioration consistent with the development of septic shock, or a structural issue such as there being a general lack of ICU beds to accommodate these patients. Additional sub-studies such as ED occupancy (i.e. overcrowding), the number of wards and number of beds that a patient is transferred between (i.e. patient flow path) and availability of diagnostic procedures should be investigated to provide additional insights into the process and structure of how patients enter and navigate through the hospital.

## Abbreviations

ACCS: Ambulatory care classification system
ADT: Admission, discharge, transfer
AHS: Alberta health services
CTAS: Canadian triage and acuity scale
DAD: Discharge abstract database
ED: Emergency department
FMC: Foothills medical centre
ICU: Intensive care unit
IQR: Interquartile range
ODBC: Open data base connectivity
PLC: Peter Lougheed centre
RGH: Rockyview general hospital
SSC: Surviving sepsis campaign
